# Tunable Wide-Angle Tunneling in Graphene-Assisted Frustrated Total Internal Reflection

**DOI:** 10.1038/srep19975

**Published:** 2016-01-27

**Authors:** Thang Q. Tran, Sangjun Lee, Hyungjun Heo, Sangin Kim

**Affiliations:** 1 Department of Electrical and Computer Engineering, Ajou University, Suwon, South Korea; 2 Department of Energy System Research, Ajou University, Suwon 443-749, Korea

## Abstract

Electrically tunable permittivity of graphene provides an excellent tool in photonic device design. Many previous works on graphene-based photonic devices relied on variable absorption in graphene, which is naturally small in the optical region, and resonant structures to enhance it. Here we proposed a novel scheme to control evanescent coupling strength by inserting two graphene layers to a frustrated total internal reflection (FTIR) configuration. The resulting structure behaves in a drastically different way from the original FTIR: optical transmission though the structure can be electrically controlled from ~10^−5^ to ~1 with little dependency on angle of incidence. This unique feature stems from the fact that the permittivity of doped graphene can be close to zero at a certain photon energy. The electrical controllability of evanescent coupling strength can enable novel design of optical devices. As a proof-of-concept, we designed a waveguide-type optical modulator of a novel operation principle: transmission modulation depends on the electrically controlled existence of a guided-mode of the waveguide, not the variation of the ohmic loss of graphene, resulting in a low insertion loss and a small device footprint.

Recently, graphene^1^ has been the subject of intense research because of its unique electrical properties, i.e., zero band gap^2^,^3^ and high electrical mobility^4^. In addition, when graphene is electrically or chemically doped, its optical properties drastically change from dielectric-like to metal-like^5^. This behavior has been utilized in many novel tunable optical nanostructures such as an optical switch^5^, metamaterials^6^, a polarizer^7^, an antenna^8^, photodetectors^9^,^10^, and sensors^11^. However, monolayer graphene, which is one atom thick, is limited in its ability to interact with light^12^. Therefore, to enhance light-matter interaction in graphene, it is necessary to employ resonant nanostructures such as a photonic crystal structure^13^,^14^, a patterned surface^6^,^15^, or nanoparticles^16^.

Among the many graphene-based optical devices, optical modulators have attracted much interest because of their broad optical bandwidth and Si waveguide compatibility^5^. In most of the previously proposed graphene-based modulators, variable ohmic loss was used to modulate optical transmission^5^,^6^,^14^,^17^, and low field confinement in an extremely thin graphene layer required a long device because resonant nanostructures can be hardly used in waveguide-type graphene-based modulators.

In this work, we propose a novel scheme to modulate optical transmission by combining the tunable permittivity of graphene, which can be very close to zero, and the frustrated total internal reflection (FTIR)^18^. FTIR is a well-known phenomenon studied by Newton, Fresnel, and others^19^, and has been used in various applications such as Kretschmann and Otto configurations in surface plasmon coupling^20^ and macroscopic multitouch-sensing devices^21^. In our proposed scheme, two graphene layers are added to the FTIR structure, and the optical transmission of the structure drastically changes when the permittivity of the graphene layers is tuned about a minimum magnitude point. The operating principle of the scheme is analyzed with respect to the dispersion characteristics of leaky modes supported by the proposed graphene-assisted (GA)-FTIR structure. In addition, we present our design of a waveguide-type optical modulator based on the proposed scheme as an example of its many possible applications. Because the GA-FTIR scheme does not rely on the ohmic loss of graphene, an optical modulator with low insertion loss and high extinction ratio (modulation depth) can be achieved with a small device footprint of several times of an operating wavelength without any resonant nanostructure.

## Results

### GA-FTIR scheme

**Figure 1:**
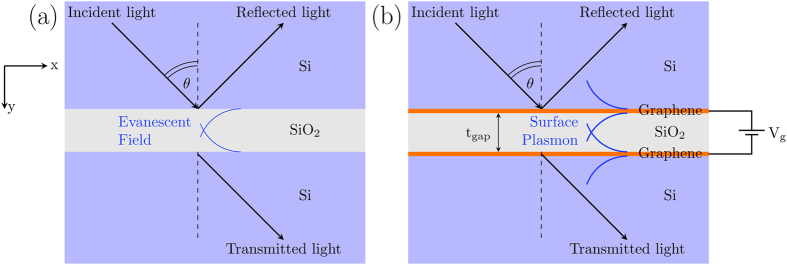
(**a**) Schematic of standard FTIR. (**b**) Schematic of GA-FTIR in which a graphene layer is inserted at both Si-SiO_2_ interfaces. In the standard FTIR scheme, when the angle of incidence is larger than the total critical angle, light tunneling can occur because of the coupling of the evanescent waves at the Si-SiO_2_ interfaces. In the GA-FTIR scheme, the inserted graphene layers drastically change the optical response of the structure.

When light from a high-index medium is refracted on the surface to a lower-index medium, total internal reflection will occur if the angle of incidence exceeds a certain limit known as the critical angle. If the low-index medium is very thin and another high-index medium is added to the end of the structure, part of the incident light can tunnel through the low-index medium and transmit to the other end; this is known as FTIR^18^. A schematic diagram of the FTIR effect is shown in Fig. 1(a). This effect is due to the coupling of the evanescent surface waves at the interface between the high- and low-index media when the angle of incidence is larger than the critical angle. This coupling allows light to bypass the optical potential barrier. The FTIR effect depends mainly on the ratio of the operating wavelength to the thickness of the low-index layer; the transmission exponentially decreases as the ratio increases. The tunneling efficiency in FTIR can in theory be modulated by dynamically modifying the thickness of the layer or the index of the low-index layer, which is extremely difficult to realize. An alternative way to modulate the behavior of FTIR is to introduce an additional tunable coupling mechanism. Our proposal is illustrated in Fig. 1(b) and is called graphene assisted FTIR, or GA-FTIR, whereby a graphene layer is inserted at each interface between the high- and low-index media. The graphene layers provide a plasmonic mode if the chemical potential (*E*_F_) of graphene is properly controlled to make its permittivity negative^22^. The plasmonic mode enhances the coupling and thus, the transmission. Furthermore, the permittivity of graphene can approach zero depending on *E*_F_ , yielding a reflection coefficient of approximately one, thus, greatly reducing the transmission. Because the *E*_F_ of graphene can be controlled by applying a gate voltage^5^, the transmission of the GA-FTIR structure can be modulated electrically.

### Tunable tunneling behavior of a GA-FTIR scheme

**Figure 2:**
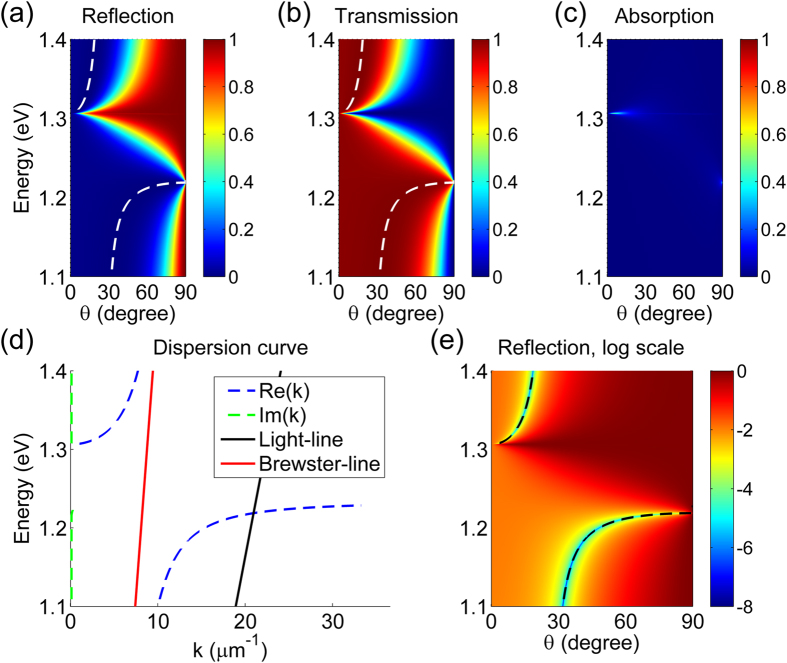
Characteristics of the GA-FTIR structure with *t*_gap_ = 10 nm and *E*_F_ = 0.9 eV. (**a**) Reflection, (**b**) transmission, and (**c**) absorption versus angle of incidence *θ* of the structure under TM illumination, numerically calculated using the RCWA method. (**d**) Dispersion relationship of the structure. (**e**) Reflection of the structure on a logarithmic scale versus angle of incidence. The dashed curves in (**a,b**) represent the photon energies of the minimum reflection and the maximum transmission, respectively. The black dashed lines in (**e**) correspond to the phase-matching condition of the dispersion curve. There are two opposite effects with respect to the angle of incidence: high reflection over a wide angle range at *E*_ph_ = 1.304 eV and high transmission over a wide angle range at *E*_ph_ = 1.215 eV.

Figure 2(a,c) show, respectively, the calculated reflection, transmission, and absorption spectra of the GA-FTIR structure under transverse magnetic (TM) illumination with various angle of incidences. We assumed that *E*_F_ = 0.9 eV and the thickness of the SiO_2_ layer *t*_gap_, was 10 nm. The spectra were calculated using the rigorous coupled-wave analysis (RCWA) method. As expected, the presence of graphene drastically modified the behavior of the FTIR. At the photon energy *E*_ph_ of 1.304 eV (λ = 951 nm), reflection is approximately 100% over a wide incident angle range. This is attributed to the near-zero permittivity of graphene at that photon energy when *E*_F_ = 0.9 eV, which is the result of the light incident at an oblique angle on the epsilon-near-zero (ENZ) material, as previously reported^23^. In addition, when *E*_ph_ ~ 1.215 eV (corresponding to λ = 1021 nm), transmission is high with little dependence on angle over a wide incident angle range. Figure 2(c) shows that there is little optical loss in the GA-FTIR structure.

To understand the behavior of the GA-FTIR, we calculated the dispersion curves of leaky modes in the GA-FTIR structure using the method previously described by J. Hu and C. R. Menyuk^24^. (See Section 1 in the Supplementary Materials online for details on the dispersion calculation.) The dispersion curves in Fig. 2(d) exhibit two leaky mode regions. In one region for *E*_ph_ > 1.304 eV, the real part of the permittivity of graphene is positive and approaches zero at *E*_ph_ = 1.304 eV. This region is known as the locus of Brewster angle in the (*w*, *k*) plane^25^. In the second region for *E*_ph_ < 1.215 eV, there is the leaky plasmonic mode due to the two graphene layers. These leaky modes can be excited by the plane wave incident from the high-index medium (Si) because their dispersion curves lie above the light line (black solid line). Using the dispersion curves of the leaky modes, we calculated the *E*_ph_ that satisfies the phase-matching condition as a function of the angle of incidence. The plots are shown in Fig. 2(e) (black dashed curves) which also shows the reflection spectrum of Fig. 2(a) on a logarithmic scale for comparison. The phase-matching condition curves coincide exactly with the loci of the minimum (near-zero) reflection. The dispersion curve of the second leaky mode (*E*_ph_ < 1.215 eV) is very flat near the light line due to the thinness of the graphene layer^26^. Because of this flat dispersion characteristic, the high transmission at *E*_ph_ ~ 1.215 eV has little dependence on the angle of incidence over a wide angle range. Figure 2(d) shows that the imaginary part of *k* for the two leaky modes is near zero, which explains why the optical loss of the GA-FTIR structure is so small, as seen in Fig. 2(c).

**Figure 3:**
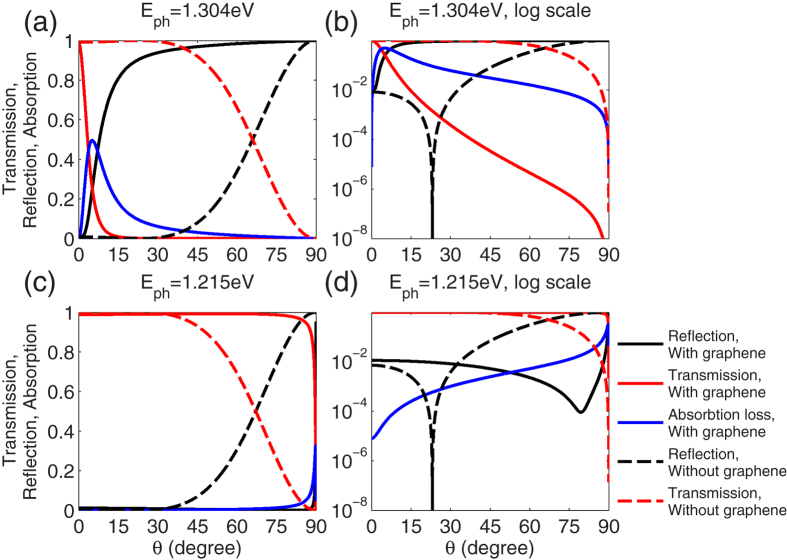
Wide-angle performance of the GA-FTIR structure with *t*_gap_ = 10 nm and *E*_F_ = 0.9 eV. Dashed lines represent the performance of the original FTIR structure without the two graphene sheets. (**a,b**) The transmission, reflection, and absorption of the structure at *E*_ph_ = 1.304 eV. (**c,d**) The transmission, reflection, and absorption of the structure at *E*_ph_ = 1.215 eV. (**a,c**) are on a normal scale and (**b,d**) are on a logarithmic scale. Note the GA-FTIR shows high-reflection at *E*_ph_ = 1.304 eV and low-reflection at *E*_ph_ = 1.215 eV over a wide range of *θ*.

To illustrate the unique performance of the GA-FTIR structure for a wide range of angle of incidence, the reflection and transmission spectra for *E*_ph_ = 1.304 and 1.215 eV are presented in Fig. 3, along with the spectral-angular performances of the original FTIR structure. For the photon energy of ENZ, *E*_ph_ = 1.304 eV, transmission of <1% was obtained for angle of incidence *θ* > 15°, as demonstrated in Fig. 3(a,b). Reflection >80% was obtained over the same range of angle of incidence. The ohmic loss of graphene is responsible for the lowered reflection. At *E*_ph_ = 1.215 eV, transmission >98.5% was observed over an angle range of 0^°^ < *θ* < 75°, where reflection and absorption were both <1% as shown in Fig. 3(c,d). Therefore, the two graphene layers greatly modified the spectral-angular performances of the original FTIR structure.

**Figure 4:**
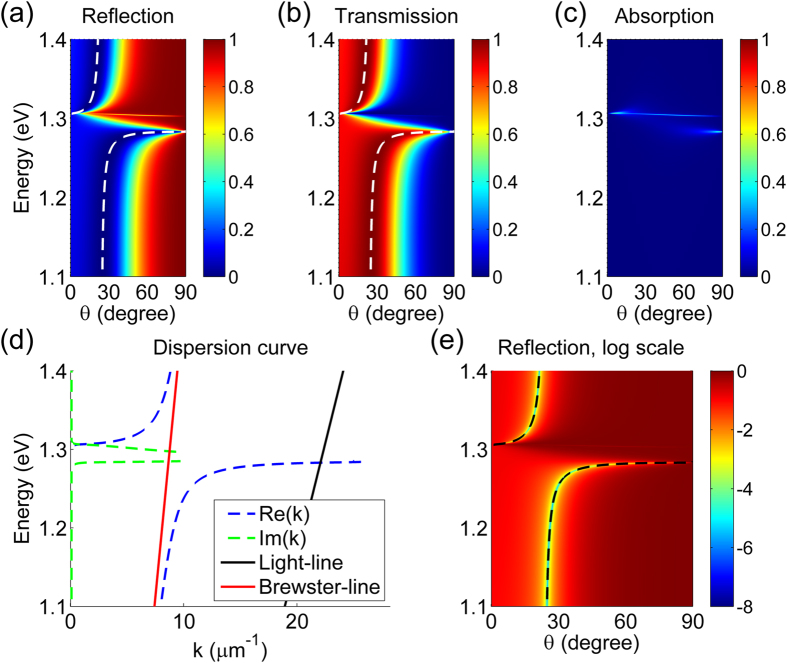
Characteristics of the GA-FTIR structure with *t*_gap_ = 40 nm and *E*_F_ = 0.9 eV. (**a**) Reflection, (**b**) transmission, and (**c**) absorption versus angle of incidence *θ* of the structure under TM illumination, numerically calculated using the RCWA method. (**d**) Dispersion relationship of the structure. (**e**) Reflection of the structure on a logarithmic scale versus angle of incidence. The dashed curves in (**a,b**) represent the photon energies of the minimum reflection and the maximum transmission, respectively. The black dashed lines in (**e**) correspond to the phase-matching condition of the dispersion curve.

Figure 4 shows the reflection, transmission, and absorption spectra and the dispersion curve of the GA-FTIR structure with *t*_gap_ = 40 nm but the same chemical potential used in the previous calculations (*E*_F_ = 0.9 eV). The behavior of the structure is similar to that with *t*_gap_ = 10 nm. High reflection occurred at the same photon energy, *E*_ph_ = 1.304 eV, as that for the structure with *t*_gap_ = 10 nm because *E*_F_ was the same. However, the photon energy for high-transmission shifted closer to that of the ENZ point (*E*_ph_ = 1.283 eV) because of the corresponding shift of the second leaky mode in the dispersion curve, as seen in Fig. 4(d). The phase-matching condition calculated using the dispersion curve agrees well with the locus of the minimum reflection, as seen in Fig. 4(e). If we want to modulate the transmission of the GA-FTIR structure by controlling the chemical potential of graphene, a smaller photon energy difference between the high reflection point (the ENZ point) and the low reflection point is beneficial, as discussed below.

**Figure 5:**
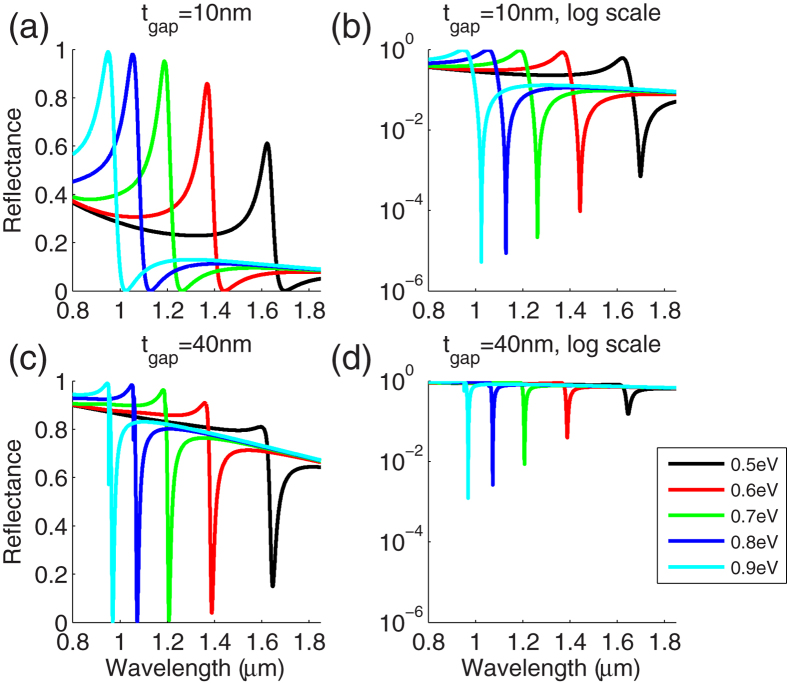
Performance of the tunable reflectance of the GA-FTIR structure at *θ* = 60° with different *E*_F_. (**a,b**) Reflectance of the structure with *t*_gap_ = 10 nm. (**c,d**) Reflectance of the structure with *t*_gap_ = 40 nm. (**a,c**) are linear scale and (**b,d**) are logarithmic scale.

We investigated the tunability of the spectral behavior of the GA-FTIR. Figure 5 shows the reflectance spectra of the GA-FTIR structure with *t*_gap_ = 10 and 40 nm and different chemical potentials of graphene but with a fixed angle of incidence of *θ* = 60°. The choice of the angle of incidence was arbitrary. Changing *E*_F_ shifts the wavelength (i.e., photon energy) at which the minimum (near-zero) permittivity of graphene occurs and thus, the wavelength of the maximum reflectance (*R*_max_). Similarly, the wavelength of the edge of the second leaky mode, where the minimum reflectance (*R*_min_) occurs, also shifts with *E*_F_. Therefore, the reflectance of the GA-FTIR at a certain wavelength can be modulated by electrically controlling *E*_F_ . *R*_max_ tends to increase with *E*_F_ because the minimum permittivity of graphene tends to zero as *E*_F_ increases, according to the Kubo formula^27^. As the minimum permittivity nears zero, the imaginary part of the permittivity value decreases, which causes the decrease in the loss of the leaky mode. Thus, *R*_min_ tends to decrease as *E*_F_ increases. Consequently, the modulation depth, defined as *R*_max_/*R*_min_, increases as *E*_F_ increases. For the structure where *t*_gap_ = 10 nm, shown in Fig. 5(a,b), *R*_max_ ~ 98.9% and *R*_min_ ≤ 10^−5^ are achieved at *E*_F_ = 0.9 eV. In this case, the ratio of the modulation depth to the insertion loss, which is frequently used as a figure of merit (FOM) for modulators, is ~1040. To the best of our knowledge of graphene-based modulators, this FOM is higher than the best value reported in the literature^28^,^29^.

Changing the gap size does not fundamentally alter the behavior of the structure. For the structure where *t*_gap_ = 40 nm, shown in Fig. 5(c,d), the reflectance in general is higher than that of the structure with *t*_gap_ = 10 nm because of the wider gap. On the other hand, the change in chemical potential (Δ*E*_F_) that is required for full modulation of reflectance is smaller because the difference in photon energy between the ENZ point and the second leaky mode edge is smaller, as mentioned above. For example, if the operating wavelength is λ = 951 nm, then Δ*E*_F_ = 0.07 eV (from 0.9 to 0.97 eV) is needed for the full modulation of reflectance when *t*_gap_ = 10 nm. Whereas, a smaller Δ*E*_F_ , e.g., Δ*E*_F_ = 0.02 eV (from 0.9 to 0.92 eV), is required for the full modulation of reflectance when *t*_gap_ = 40 nm.

### Waveguide-type optical modulator based on a GA-FTIR structure

**Figure 6:**
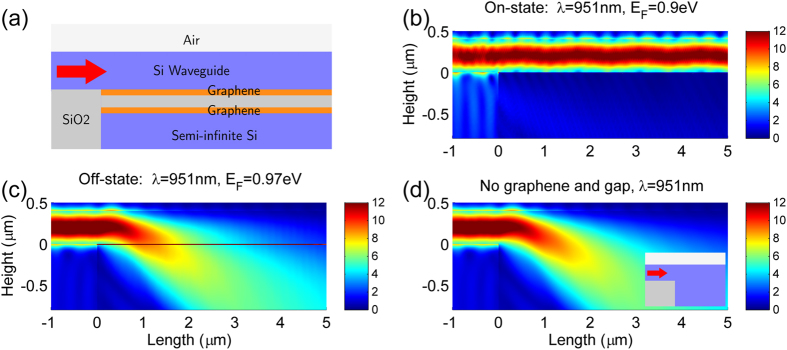
A slab waveguide-type modulator based on the GA-FTIR. (**a**) Schematic diagram of the modulator in which one of the semi-infinite high-index (Si) media in the GA-FTIR structure is replaced by a 400-nm-thick single-mode slab waveguide, the two graphene layers are separated by a 10-nm-thick SiO_2_ layer, and a semi-infinite Si layer at the bottom serves as a leaky channel. The red arrow indicates the light input. (**b,c**) Electric field distributions obtained from 2D FEM simulation of the device in the on-state (*E*_F_ = 0.9 eV) and the off-state (*E*_F_ = 0.97 eV), respectively, at λ = 951 nm. (**d**) Electric field distribution from 2D FEM simulation of the device, with the graphene/SiO_2_/graphene channel removed, as shown in the inset. The 2D wave propagations in (**c,d**) are almost identical. This demonstrates the abnormal wide-angle transparency of the proposed structure, even in the presence of ohmic loss in graphene.

In this work, we propose a waveguide-type optical modulator based on the GA-FTIR scheme. The wide-angle reflectance modulation properties of the GA-FTIR structure can be utilized to control the existence of a guided mode if one of the two semi-infinite high-index (Si) media is replaced with a slab waveguide, as depicted in Fig. 6(a). The proposed modulator has a 400-nm-thick Si layer that acts as a single-mode slab waveguide, two graphene layers separated by a 10-nm-thick SiO_2_ layer, and a semi-infinite Si medium on the bottom. Figure 6(b) shows a two-dimensional (2D) finite-element method (FEM) simulation, performed using COMSOL, of TM wave propagation in the proposed structure, with *E*_F_ = 0.9 eV and an operating wavelength of 951 nm. The operating wavelength and *E*_F_ were chosen to obtain wide-angle high reflectance from the GA-FTIR scheme. As a result, the top slab supports the guided mode, and thus a launched TM wave propagates along the top slab waveguide, resulting in a high-transmission state (on-state). Figure 6(c) shows the TM wave propagation for *E*_F_ = 0.97 eV at the same operating wavelength. In this case, the GA-FTIR scheme offers very low reflectance over a wide range of angle of incidences and, thus, the top slab no longer supports the guided mode, so a launched wave leaks into the bottom Si medium, resulting in a low-transmission state (off-state). For comparison, TM wave propagation in a structure in which the two graphene layers and the SiO_2_ gap are replaced by a Si medium, as shown in the inset of Fig. 6(d), was also calculated. The wave propagation shown in Fig. 6(d) is nearly identical to the off-state case shown in Fig. 6(c). This implies that the enhanced wide-angle high transmission (low reflectance) of the GA-FTIR with assisted tunneling is very similar to that of a transparent medium. To our knowledge, this wide-angle tunable transparency, even with the presence of ohmic loss in graphene, is unique to this particular structure.

**Figure 7:**
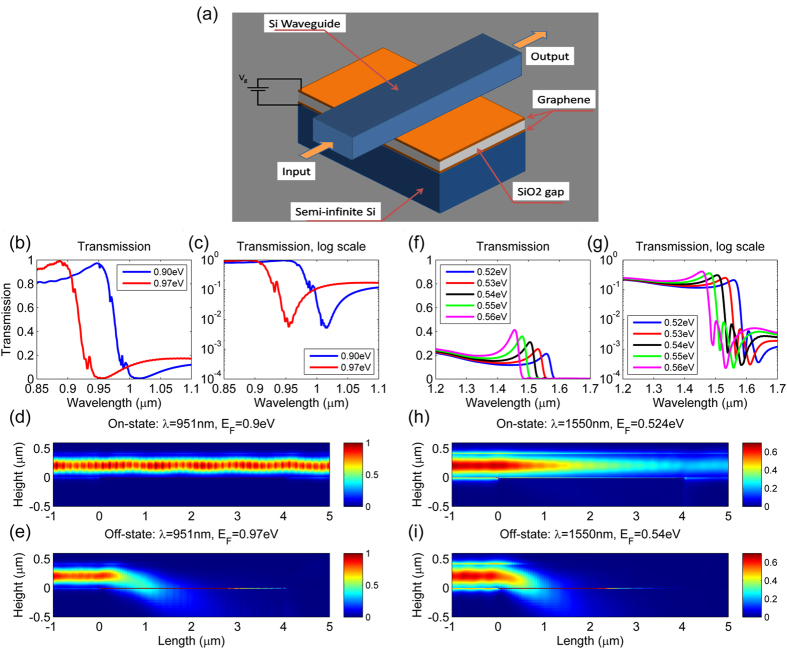
3D FDTD simulation of a waveguide-type modulator based on the GA-FTIR. (**a**) Schematic of the proposed device, in which the SiO_2_ gap size is *t*_gap_ = 10 nm, the Si optical waveguide is 400 nm high and 500 nm wide, and the device is 4 μm long. (**b**,**c**) Transmission of the device with *E*_F_ = 0.9 and 0.97 eV, on linear and log scales, respectively. (**d**,**e**) Field intensity profiles at λ = 951 nm in the on-state (*E*_F_ = 0.90 eV) and the off-state (*E*_F_ = 0.97 eV), respectively. (**f**,**g**) Transmission of the device for five chemical potentials from 0.52 to 0.56 eV, on linear and log scales, respectively. (**h**,**i**) Intensity profiles at λ = 1550 nm in the on-state (*E*_F_ = 0.524 eV) and the off-state (*E*_F_ = 0.54 eV), respectively. The relatively high insertion loss of the device at λ = 1550 nm (*E*_F_ = 0.524 eV) is due to the low reflectance of GA-FTIR at the ENZ point of graphene. The reflectance can be improved by employing thicker (multilayer) graphene, as discussed in Section 2 of the Supplementary Information available online.

Building on the results of the 2D optical modulator calculations, we investigated a more practical device structure, shown in Fig. 7(a), and performed three-dimensional (3D) finite difference time domain (FDTD) calculations using commercial software (Lumerical). In all cases, the SiO_2_ layer was 10 nm thick, the Si optical waveguide was 400 nm high and 500 nm wide, and the device was 4 μm long. Figure 7(b) shows the transmission spectra with *E*_F_ = 0.9 and 0.97 eV, and Fig. 7(c) shows the spectra on a logarithmic scale. The figures show full transmission modulation from on-state to off-state at λ = 951 nm, as was observed in the 2D calculations. Figure 7(d,e) show the field intensity profiles of the on-state and the off-state, respectively, at λ = 951 nm. The device had a very low insertion loss and very high extinction ratio, while being compact. The 4-μm-long device can achieve a strong switching performance. The length of the device determines the transmission in the off-state, so the device can be even shorter if we can compromise on the modulation depth.

The modulation performance of the device depicted in Fig. 7(a) was numerically investigated using lower chemical potential values for graphene (i.e., *E*_F_ = 0.524 and 0.54 eV), which are appropriate for an operating wavelength of 1550 nm. Figure 7(f,g) present the transmission spectra and Fig. 7(h,i) show the field intensity profiles of the on-state and off-state, respectively, of this device. The insertion loss is much higher, which is consistent with the results obtained from the RCWA calculations shown in Fig. 5(a,b). This low transmission is due to the relatively weak ENZ effect of graphene at the longer wavelength; the reflectance of the GA-FTIR is not high enough. Therefore, if the number of graphene layers is increased, then the reflectance of the GA-FTIR will increase, thus achieving higher transmission by the modulator. At λ = 1550 nm, a three-graphene-layer device obtained an insertion loss of ~1 dB (please refer to Section 2 in the Supplementary Materials online for numerical results). An important feature of the proposed modulator is its wide optical bandwidth. Without changing any design parameters, as long as the top waveguide remains a single-mode operation, one can obtain a wide range of operating wavelengths by choosing the appropriate chemical potential of graphene via gate voltage.

## Discussion

In this work, we proposed a novel scheme to control optical tunneling in a FTIR configuration by introducing two graphene layers doped to have the ENZ effect. In addition to the incident angle independent high reflection due to the ENZ graphene layer, very flat dispersion characteristics of the leaky mode supported by two plasmonic coupled graphene layers provides strong optical tunneling modulation over a wide incident angle range, without requiring any complex resonant structures. The electrical controllability of evanescent coupling strength provided by the proposed scheme, which is unique to this scheme to the best of our knowledge, can enable novel design of optical devices. As a proof-of-concept, we designed a waveguide-type optical modulator of a novel operation principle: transmission modulation depends on the electrically controlled existence of a guided-mode of the waveguide.

The operating wavelength of the proposed scheme can be varied over a wide range by changing the chemical potential of graphene, E_F_ . In practice, the breakdown of a gap material, SiO_2_ in the gate structure limits the maximum achievable value of E_F_: the carrier density in graphene is related to the chemical potential E_F_ as *N*_*G*_ = E_F_^2^/(*πħ*^*2*^*V*_F_^*2*^), where *ħ* is the reduced Plank constant and *V*_F_ is the Fermi velocity, and from the simple capacitive model, the electric field in the gap layer is given by *qN*_*G*_*/ε*_*o*_*ε*_*SiO2*_ , which should be smaller than a certain value to avoid the breakdown of the gap material. This limitation can be overcome with combining chemical doping and electrostatic doping induced by a gate voltage. Chemical doping of up to E_F_ = 2.0 eV have been successfully demonstrated^30^. For example, to achieve a chemical potential variation from E_F_ = 0.9 to 0.97 eV, we can dope graphene chemically up to E_F_ = 0.9 eV and apply varying gate electric field from 0 to ~8.3 MV/cm (assuming *V*_F_ = 10^6^ m·s^−1^), which lies within SiO_2_ breakdown electric field of ~13 MV/cm^31^.

The use of a better insulator with higher permittivity and breakdown electric field such as Al_2_O_3_ could increase the maximum achievable chemical potential. The relative permittivity of Al_2_O_3_ is 1.75^2^ and the breakdown electric field of high quality Al_2_O_3_ film formed by the atomic layer deposition can be as high as 30 MV/cm^32^. With Al_2_O_3_ adopted for the gap material, E_F_ up to ~0.83 eV can be achieved by electrostatic doping alone, induced by a gate voltage, which is much higher than the required value of E_F_ for device operation at λ = 1550 nm and will allow the operating wavelength as short as ~1182 nm.

Another way to achieve higher chemical potential for a given gate voltage is to increase the Fermi velocity: a two fold increase in Fermi velocity would reduce the required gate voltage by a factor of four to achieve the same chemical potential. The value of *V*_F_ =10^6^ m·s^−1^ used for the calculation in this work is a conservative choice and likely represents the lower limit, corresponding to the case where electron - electron interactions are weak^33^. Recently, it was experimentally demonstrated that Fermi velocity of graphene could be engineered by substrate modification, and the values as high as *V*_F_ ~ 2.49 × 10^6^ m·s^−1^ and ~1.49 × 10^6^ m·s^−1^ were achieved with quartz and BN substrates, respectively^34^. Thus, proper choice of the high-index material in the GA-FTIR scheme can yield larger Fermi velocity. If the graphene layers have Fermi velocity larger than *V*_F_ ~ 1.19 × 10^6^ m·s^−1^, electrostatic doping alone can achieve E_F_ ~ 0.54 eV and enable device operation at λ =  1550 nm, avoiding breakdown of SiO_2_.

As mentioned early, the operating wavelength of the proposed scheme can be varied over a wide range and the allowed shortest operating wavelength is limited only by the maximum achievable chemical potential of graphene. For the GA-FTIR scheme with SiO_2_ gap material, the maximum achievable chemical potential by the gate voltage is ~0.45 eV even for the conservative value of Fermi velocity of *V*_F_ = 10^6^ m·s^−1^, which is large enough to allow device operation at λ = ~2 μm.

## Methods

In our numerical analysis, the permittivity of Si and SiO_2_ were assumed to be 3.4^2^ and 1.45^2^, respectively, and the permittivity of graphene was calculated using the Kubo formula^27^ with following assumptions: the thickness of graphene was 1 nm; the Fermi velocity *V*_F_ was 10^6^ m·s^−1^; and the mobility μ was 1 m^2^·V^−1^·s^−1^.

### Simulations

The spectral response of the scheme and its angle dependency were calculated by using the commercial R-Soft DFMOD RCWA software. The finite element method commercial software COMSOL was used to calculate the frequency domain field distribution of the 2D waveguide type structure. For 3D structure, the FDTD commercial package from Lumerical was used.

## Additional Information

**How to cite this article**: Tran, T. Q. *et al.* Tunable Wide-Angle Tunneling in Graphene-Assisted Frustrated Total Internal Reflection. *Sci. Rep.* **6**, 19975; doi: 10.1038/srep19975 (2016).

## Supplementary Material

Supplementary Information
