# Supplementary material for: Tunable Wide-Angle Tunneling in Graphene-Assisted Frustrated Total Internal Reflection
Source: Sci Rep. 2016 Jan 27;6:19975. doi: 10.1038/srep19975 (PMC4728486; doi:10.1038/srep19975)
Supplement: Supplementary Information [file srep19975-s1.pdf]

Supplementary Materials for "Tunable Wide-Angle Tunneling in Graphene-Assisted Frustrated Total Internal Reflection"

Thang Q. Tran, Sangjun Lee, Hyungjun Heo and Sangin Kim*
*Corresponding Author: E-mail:sangin@ajou.ac.kr
Department of Electrical and Computer Engineering, Ajou University, Suwon, South Korea

1. Dispersion relationship of leaky modes supported in GA-FTIR structure

The magnetic field of a transverse magnetic (TM) polarized mode propagating with a propagation constant β in the x direction in a multilayered structure can be written in the frequency domain as [Ref. S1]

$$H_i = H_0 f_i(y)\exp(i\beta x)e_z, \qquad \text{(Eq. S1a)}$$

where

$$f_i(y) = a_i \exp(\alpha_i[y - y_i]) + b_i \exp(-\alpha_i[y - y_i]), \qquad \text{(Eq. S1b)}$$

$$\alpha_i = (\beta^2 - \epsilon_i k_0{}^2)^{1/2}, \qquad \text{(Eq. S1c)}$$

and ε_i is the permittivity of each layer and i is a layer number (i = 1-5).

Imposition of continuity of E_x and H_z at the surfaces of the structure leads to a system of eight linear equations (two for each of the four surfaces) that incorporates four pairs of unknowns (a_i and b_i) plus the propagating constant β. To find the leaky modes, we assumed forward-propagating waves in both the first layer and the last layer, where $b_1 = b_5 = 0$. Finally, the dispersion relationship was derived from the condition that to have solutions, the determinant of the system of eight equations with nine unknowns is zero. The resulting dispersion relationship is given by

$$\begin{aligned}
&-e^{2(t_{\text{gap}}+2t_{\text{G}})\alpha_2}(u_1{}^2-u_2{}^2)(u_2-u_3)^2 + e^{2t_{\text{gap}}(\alpha_2+\alpha_3)}(u_1{}^2-u_2{}^2)(u_2-u_3)^2 \\
&-e^{2t_{\text{gap}}\alpha_2}(u_1{}^2-u_2{}^2)(u_2+u_3)^2 + e^{2(t_{\text{gap}}+2t_{\text{G}})\alpha_2+2t_{\text{gap}}\alpha_3}(u_1{}^2-u_2{}^2)(u_2+u_3)^2 \\
&-2e^{2(t_{\text{gap}}+t_{\text{G}})\alpha_2}(u_1{}^2+u_2{}^2)(u_2{}^2-u_3{}^2) + 2e^{2(t_{\text{gap}}+t_{\text{G}})\alpha_2+2t_{\text{gap}}\alpha_3}(u_1{}^2+u_2{}^2)(u_2{}^2-u_3{}^2) = 0,
\end{aligned} \qquad \text{(Eq. S2)}$$

where $u_i = \alpha_i / \epsilon_i$, i = 1,2,3, t_{gap} is the thickness of the SiO_2 layer, and t_{G} is the thickness of the graphene. To simplify (Eq. S2), we imposed the following symmetric conditions: $u_5 = u_1$, $k_5 = k_1$, $u_4 = u_2$, and $k_4 = k_2$.

The dispersion relationship was solved for a given frequency to find the propagation constant β and coefficients a_i and b_i, by which we then calculated the field distribution of the mode in the

structure. The calculation for $t_{gap} = 10$ nm, chemical potential of graphene $E_F = 0.9$ eV, and photon energy $E_{ph} = 1.215$ eV is shown in Fig. S1(a) and (b). The field distribution calculated using the dispersion relationship matches that of the numerical results obtained from RCWA simulation, which are shown in Fig. S1(c) and (d).

2. Performance of GA-FTIR with thick (multilayer) graphene

The performance of the GA-FTIR at a low chemical potential is generally worse than that at a high chemical potential because the minimum epsilon value increases as E_F decreases, i.e., the ENZ effect of graphene gets weaker at lower chemical potentials. This situation can be improved by using thicker (multilayer) graphene. Assuming that the surface conductivity for each layer of randomly stacked multilayer graphene remains unchanged [Ref. S2, Ref. S3], we calculated the performance the GA-FTIR structure with 3- and 5-nm-thick graphene. Figure S2 shows the reflectance in a GA-FTIR structure with a 45-nm-thick SiO_2 gap layer for various E_F and the two graphene thicknesses at $\theta = 60°$. In general, the maximum reflectance values are greater than those for the monolayer-graphene case shown in Fig. 5, and the performance is improved more at lower E_F .

The performance of a waveguide-type modulator based on a GA-FTIR with multilayered graphene, the structure of which is the same as that shown in Fig. 7(a), was also calculated and shown in Fig. S3. The thickness of the SiO_2 gap layer was 45 nm and the E_F values were chosen to demonstrate optical modulation at an operating wavelength of 1550 nm. There were obvious improvements in insertion loss (maximum transmission) compared to the structure with monolayer graphene, the transmission of which is shown in Fig. 7(f) and (g). In addition, the performance of the structure with 5-nm-thick graphene is slightly better than that of the structure with 3-nm-thick graphene, which suggests that there is a limit to improving performance by increasing the number of graphene layers.

References for Supplementary materials:

[Ref. S1] Hu, J. & Menyuk, C. R. Understanding Leaky Modes: Slab Waveguide Revisited. *Adv. Opt. Photon.* 1, 58–106 (2009).

[Ref. S2] Chu, H.-S. & Gan, C. H. Active plasmonic switching at mid-infrared wavelengths with graphene ribbon arrays. *Appl. Phys. Lett.* 102, 231107 (2013).

[Ref. S3] Casiraghi, C. et al. Rayleigh Imaging of Graphene and Graphene Layers. *Nano Lett.* 7, 2711–2717 (2007).

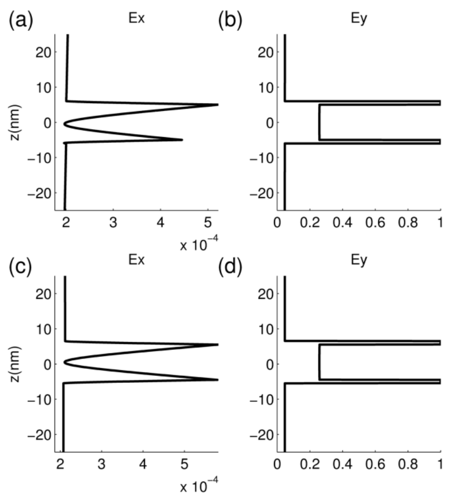

Figure S1. Field amplitude profiles calculated using the dispersion relationship: (a) E_x and (b) E_y. For comparison, field amplitude profiles from the RCWA simulation are also presented: (c) E_x and (d) E_y. The coordinate system is the same as in Figure 1. T_{gap} = 10 nm, E_F = 0.9 eV, and λ = 1022 nm (E_{ph} = 1.215 eV).

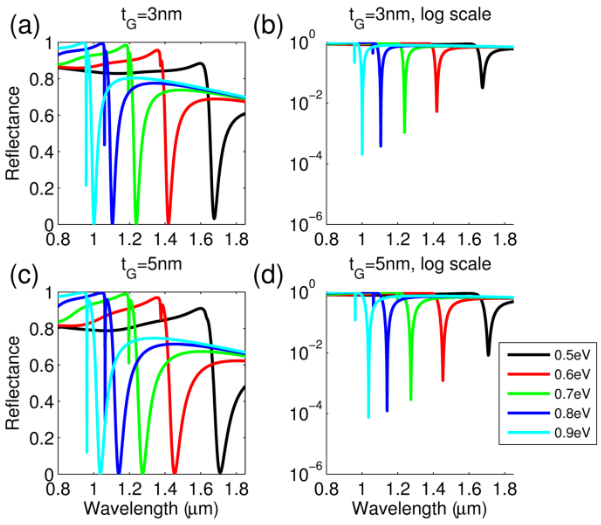

Figure S2. Effect of the thickness of graphene (i.e., the number of graphene layers) on the performance of the GA-FTIR with respect to reflectance. (a) and (b) Reflectance spectra for t_G = 3 nm. (c) and (d) Reflectance spectra for t_G = 5 nm. The SiO_2 gap thickness was 45 nm.

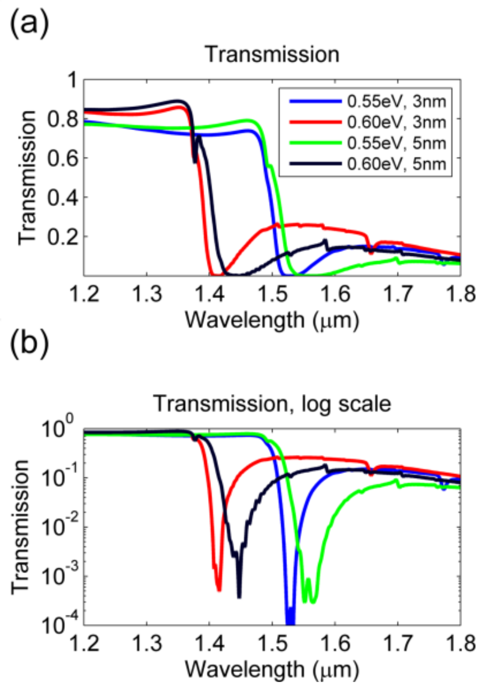

Figure S3. Transmission spectra of the waveguide-type modulator based on GA-FTIR with thick (multilayer) graphene on (a) a linear scale and (b) a log scale. The blue and the red curves are for $t_G = 3$ nm and the green and black curves are for $t_G = 3$ nm. The thickness of the SiO_2 layer was 45 nm and E_F values were chosen for an operating wavelength of 1550 nm.
